# The Effects of Carbon–Silica Dual-Phase Filler on the Crosslink Structure of Natural Rubber

**DOI:** 10.3390/polym14183897

**Published:** 2022-09-18

**Authors:** Jingyi Wang, Hongbing Jia

**Affiliations:** 1 School of New Materials and Shoes & Clothing Engineering, Liming Vocational University, Quanzhou 362000, China; 2 Key Laboratory for Soft Chemistry and Functional Materials of Ministry of Education, Nanjing University of Science and Technology, Nanjing 210094, China

**Keywords:** natural rubber, filler, CSDPF, vulcanization, network structure

## Abstract

Carbon–silica dual-phase filler (CSDPF)/natural rubber (NR) vulcanizate was prepared by mechanical blending, followed by a hot-press vulcanization. The dispersion of CSDPF in the NR matrix and the effects of CSDPF on the filler–rubber interaction and structure of the rubber network were studied. Scanning electron microscope results showed that CSDPF dispersed uniformly; however, there were some aggregates of CSDPF when loading too many fillers. With an increase in CSDPF, the interaction between CSDPF and NR chains increases, which was detected by bound rubber in the CSDPF/NR compound. The spectra of solid-state nuclear magnetic resonance revealed that CSDPF could promote the formation of poly-sulfidic crosslink in the rubber vulcanization network. Further, the molecular chain movement ability of vulcanizates decreases according to the spin–spin relaxation of ^1^H nuclei in CSDPF/NR compounds. The crosslink density of vulcanizate increases, while the chemical crosslink and physical crosslink in the vulcanization network also increase according to the tube model.

## 1. Introduction

In rubber composites, carbon black and silica are the most used fillers. For carbon black, it can effectively improve the tensile strength, tearing strength and wear resistance of the rubber material; however, it causes high friction of rubber material. In the case of silica, it can significantly reduce friction and rolling resistance of rubber material; nevertheless, the mechanical strength of silica-filled rubber should be further improved. Thus, these two kinds of fillers were often filled into rubber together in practical applications [1]. Nevertheless, because the two fillers have different surface energy and poor compatibility, they cannot form a uniform filler network in rubber materials [2], which has limitations on the improvement of rubber.

Carbon–silica dual-phase filler (CSDPF) is a kind of hybrid filler and different from the physical mixing of carbon black and silica. CSDPF is prepared through symbiosis technology in the production process of carbon black, in which the carbon black is chemically modified by organic–silicon compounds. As a result, CSDPF exhibits two kinds of phase structure, which are the carbon black phase and silica phase. In other words, carbon black and silica are doped with each other, which effectively reduces the filler–filler interaction and improves their dispersion in the rubber matrix. Wang et al. [3,4] reported a series of works about CSDPF-filled rubber, but they focused on dynamic mechanical properties, and did not systematically study the static mechanical properties, filler–rubber interaction and rubber network structure.

Vulcanization is an important process, in which the crosslink network of rubber is formed. It is known that the crosslink network significantly affects the macro-properties of the rubber. In this work, CSDPF/natural rubber (NR) vulcanizate was prepared, and the interaction between CSDPF and rubber was studied. Four methods of crosslink density, tube model, ^13^C-NMR and ^1^H-NMR were used to analyze the influence of CSDPF on the network structure of NR vulcanization.

## 2. Materials and Methods

### 2.1. Materials

CSDPF2125 (Si contained is 5.1 wt.%) was purchased from Cabot China Ltd., Shanghai. NR (RSS1) and the rest of the materials were provided by Nanjing JinSanLi Rubber & Plastic Co., Ltd., Nanjing, China.

### 2.2. Preparation of Composites

The formulation of the NR compound is summarized in Table 1. All ingredients were mixed in an LN-120 open two-roll mill (LINA machinery Industrial Co., Ltd., Dongguan China) at room temperature. The vulcanizates were prepared by a molding the above compounds at 143 °C and 15 MPa for the optimum cure time (*t*_90_).

### 2.3. Characterization

The bound rubber of compounds was determined according to Ref. [5].

(1) $$Bound\;rubber=\frac{\omega_{3}-\omega_{2}-\omega_{1}\frac{m_{1}}{m_{1}+m_{r}}}{\omega_{1}\frac{m_{r}}{m_{1}+m_{r}}\cdot \omega_{1}\frac{m_{1}}{m_{1}+m_{r}}}\times 100$$

where *ω*_1_, *ω*_2_ and *ω*_3_ are the weight of rubber before swelling, filler and the weight of rubber after drying, respectively. *m*_1_ and *m_r_* are the fraction of filler and rubber in the compound, respectively.

The tensile tests were measured on a universal testing machine (Shenzhen SANS Co., Ltd., Shenzhen, China) at a crosshead speed of 10 mm/min according to ASTM D412. The results were averaged based on five measurements.

The crosslink densities of the composites determined according to Ref. [5]. The samples were cut into rectangles (10 × 10 × 2 mm^3^) and weighed before and after being soaked in toluene for 7 days, which ensured a swelling equilibrium. The crosslink density (XLD) was calculated according to

(2) $$XLD=-\frac{\ln(1-\phi_{r})+\phi_{r}+\chi_{1}\phi_{r}^{2}}{\phi_{r}\left(\phi_{r}^{\frac{1}{3}}-\phi_{r}/2\right)}$$

(3) $$\phi_{r}=\frac{\frac{\omega_{1}}{\rho_{d}}}{\frac{n_{2}-n_{1}}{\rho_{s}}+\frac{n_{1}}{\rho_{d}}}$$

where *n*_1_ and *n*_2_ are the mass of sample before swelling and swollen, respectively. The *ρ_d_* is the density of the sample before swelling and *ρ_s_* is the toluene density (0.8669 g/mL). $\chi_{1}$ is the Flory–Huggins interaction parameter between toluene and rubber (0.391).

The freeze-fracture surface morphology of the samples was observed with a JSM-6700F scanning electron microscope (SEM, JEOL Ltd., Akishima, Japan).

Solid-state nuclear magnetic resonance (NMR) spectroscopy was performed with a Bruker Avance III NMR spectrometer (Bruker Corporation, Billerica, MA, USA) operating at 400 MHz and 100 MHz for ^1^H and ^13^C, respectively. The magic-angle spin (MAS) rate of the sample tube for the NMR measurement was 10 kHz. The spectra were recorded from a single-pulse experiment using high-power decoupling. The π/2 pulse width for ^13^C was 6 μs with a 3 μs width decoupling pulse for ^1^H. The number of scans for acquisition of spectra was 30 k. The spin–spin relaxation time (*T*_2_) were measured by the Carr–Purcell–Meiboom–Gill (CPMG) method.

## 3. Results

### 3.1. Dispersion of CSDPF

**Figure 1:**
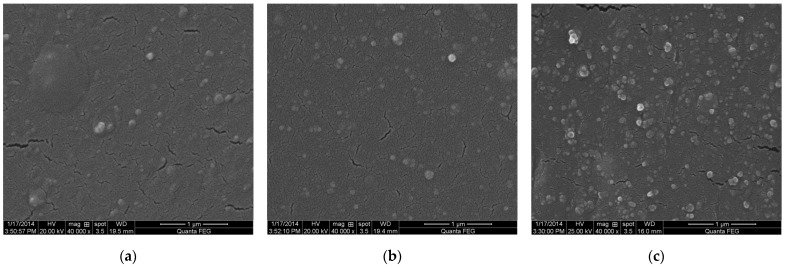
SEM images of freeze-fractured surface of CSDPF/NR vulcanizates, (**a**) C10, (**b**) C30 and (**c**) C50.

Figure 1 shows the fracture surfaces of CSDPF/NR vulcanizates. As can be seen in Figure 1a, CSDPF is evenly dispersed in the NR matrix when the filling amount is less; the larger-size CSDPF is shown on the fracture surface when the loading is increased to 50 phr (Figure 1c). Due to the high surface area (171 m^2^/g) of CSDPF, NR chains are adsorbed strongly onto CSDPF’s surface. Thus, high surface area and high loading of filler used in NR induce small distances between reinforcing fillers so that almost any rubber chain contacts at least one filler aggregate [6]. In addition, because the statistical size of polymeric chains is in the range of interaggregate distances, close-neighboring objects are probably bound together by chains adsorbed onto both aggregates. Therefore, the larger-size filler is present in SEM.

**Figure 2:**
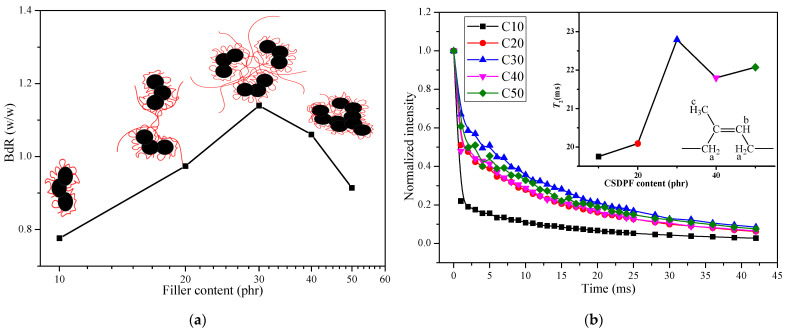
(**a**) BdR of CSDPF/NR compounds, (**b**) Spin–spin relaxation and *T*_2_ of Hb nuclei (inset image) in bound rubber.

In order to further understand the structure of filler network, bound rubber (BdR) of CSDPF/NR compound was measured and is shown in Figure 2a. Bound rubber is a part of rubber that cannot be extracted by a good solvent. From the phenomenological point of view, the bound rubber can be understood as a part of the rubber that the filler particles form into a three-dimensional reticular formation in rubber to adsorb or encapsulate in rubber [7]. Therefore, as seen in Figure 2a, the BdR increases gradually and the reaches the maximum when loading 30 phr of CSDPF. When the content of filler is low, the discrete CSDPF adsorbed a certain number of NR chain segments (such as Figure 2a illustrates). With an increase in filler, the CSDPF aggregates begin to approach each other; therefore, in addition to the NR chain segments adsorbed on the surface of the CSDPF aggregate, there are other NR chains entangled between the adjacent CSDPF aggregates. As a consequence, the BdR increases. As the amount of filler is further increased to 30 phr, more and more NR chain segments are entangled between adjacent CSDPF aggregates and there are even NR chain segments trapped between CSDPF aggregates [2,8]; thus, the BdR reaches its peak. However, when more fillers are added, the CSDPF aggregates form an agglomeration; thus, the NR segment of the adsorbed was reduced (graph embedded in Figure 2a), causing a reduction in BdR.

Figure 2b shows the spin–spin relaxation and the contact time *T*_2_ of Hb protons in CSDPF/NR vulcanizates. *T*_2_ reflects the movement of all molecules in the entire rubber network, including information of fast motion and slow motion [9]. It can be seen in Figure 2b that the *T*_2_ of the Hb protons in bound rubber increases with the addition of CSDPF, and reaches a maximum value when adding 30 phr of CSDPF. This is consistent with the trend of BdR. Because BdR increases gradually with an increase in the amount of filler; that is, the number of rubber chain segments adsorbed by each filler increases. As a result, the effect of each filler on the molecule movement of the whole rubber network of bound rubber is gradually reduced; thus, the *T*_2_ increases.

### 3.2. Crosslink Structure of Vulcanization Network

**Figure 3:**
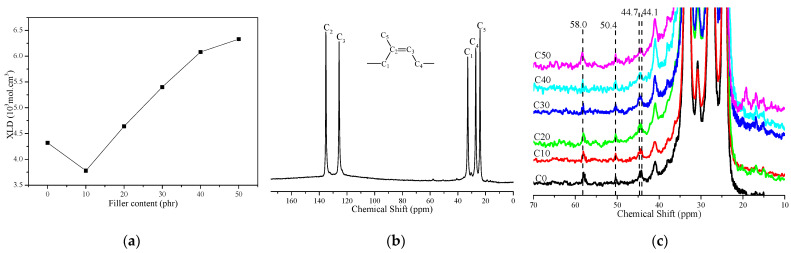
(**a**) Crosslink density (XLD), (**b**) full region of ^13^C-NMR spectra and (**c**) region from 70–10 ppm of ^13^C-NMR spectra of CSDPF/NR vulcanizates.

Figure 3a is a crosslink density (XLD) diagram of CSDPF/NR vulcanizate. It can be seen that the XLD of the vulcanizate drops when a small amount of CSDPF is added. This is due to the adsorption of accelerators on the silica phase on CSDPF, which leads to a decrease in the crosslink degree of the vulcanizate [4,10]. The XLD of the vulcanizate increases with the further addition of CSDPF. On the one hand, due to the addition of more CSDPF, the interaction between silica phases on the CSDPF surface reduced the adsorption of accelerant to a certain extent; that is, it promoted an increase in crosslink degree of vulcanizate; on the other hand, the calculation of XLD is based on the Flory–Rehner swelling model: the smaller the swelling degree of the vulcanizate is, the greater the XLD is, but, in the calculation of the swelling model, only the volume of the filler is simply deducted, and the effect of the filler–rubber interaction on the swelling volume is not taken into account [11].

In order to study the effect of CSDPF on the vulcanization crosslinking point of natural rubber vulcanizate, the solid-state ^13^C-NMR was used. Figure 3b shows a ^13^C-NMR diagram of typical natural rubber vulcanizate; Figure 3c is a ^13^C-NMR diagram of different CSDPF/NR in the 10–70 ppm region. In Figure 3b, there are five major nuclear magnetic signals that correspond to five different carbon atoms on the NR molecular chain (inset image). In Figure 3c, there are four smaller signals, and their chemical shifts are 44.1, 44.7, 50.4 and 58.0 ppm, respectively. Among them, the chemical shift of 44.7 ppm corresponds to the mono-sulfidic crosslink in the crosslinked bond, and the signals of 44.1, 50.4 and 58.0 ppm correspond to the poly-sulfidic crosslink in the crosslinked bond, respectively [12]. The signal (C5, 24.0 ppm) of methyl carbon was selected to normalize the four smaller signal intensities:

(4) $$\chi_{Xp.p.m.}=\frac{I_{Xp.p.m.}}{I_{24.0p.p.m.}}$$

Among them, *χ* represents the chemical shift of the corresponding signal peak, and *I* is the intensity of the corresponding signal peak. The results are brought into the next formula and the content distribution of the mono-sulfidic crosslink and poly-sulfidic crosslink is calculated:

(5) $$A_{Y}(\%)=\frac{\chi_{Y}}{\chi_{mono}+\chi_{poly}}\times 100$$

Among which, *Y* is the type of crosslinked bond and *χ* is the relative intensity of the signal of the corresponding crosslinked bond after normalization. The results are listed in Table 2. It can be seen that the content of the poly-sulfidic crosslink of C10 increases from 72.8% to 74.1%. In the vulcanization process, the content of the poly-sulfide bond is proportional to the ratio of sulfur/accelerator [12]. The silica phase on CSDPF will adsorb accelerators [4,10] and reduce the content of accelerators. Thus, the ratio of sulfur/accelerator is increased and, finally, the content of the poly-sulfidic crosslink in C10 is increased. With the further addition of CSDPF, the interaction between silica phases on the CSDPF surface reduces the adsorption of accelerators. As a result, the ratio of sulfur/accelerator decreases, causing a reduction in the content of the poly-sulfidic crosslink.

### 3.3. Network Structure of CSDPF/NR

The network structure of rubber includes chemical crosslink and physical crosslink, which play an important role in the mechanical properties of rubber. In this section, a tensile test performed at low strain rate, i.e., 10 mm/min, was utilized in the tube model analyses. The relevant equations are as follows [13]:

(6) $$\sigma_{M}(\lambda^{\prime })=\frac{\sigma }{\left(\lambda^{\prime }-\lambda^{\prime }{}^{-2}\right)}=G_{c}(\varphi )+G_{e}(\varphi )f(\lambda^{\prime })$$

(7) $$f(\lambda^{\prime })=2(\frac{\lambda^{\prime }{}^{0.5}-\lambda^{\prime }{}^{-1}}{\lambda^{\prime }{}^{2}-\lambda^{\prime }{}^{-1}}),\lambda^{\prime }\neq 1$$

(8) $$f(\lambda^{\prime })=1,\lambda^{\prime }=1$$

(9) $$\lambda \prime =(\lambda -1)E/E_{0}+1$$

Among them, *σ_M_* is the Mooney stress, *σ* is the actual stress, *λ*′ is the internal strain rate, *φ* is the volume fraction of the filler, *G_c_* is the contribution of the chemical crosslink to the elastic modulus, *G_e_* is the contribution of the physical crosslink to the elastic modulus, *λ* is the macro-strain ratio and *f*(*λ*) is the strain equation. *E* and *E*_0_ are the initial elastic modulus of vulcanizate with fillers and no fillers, respectively.

**Figure 4:**
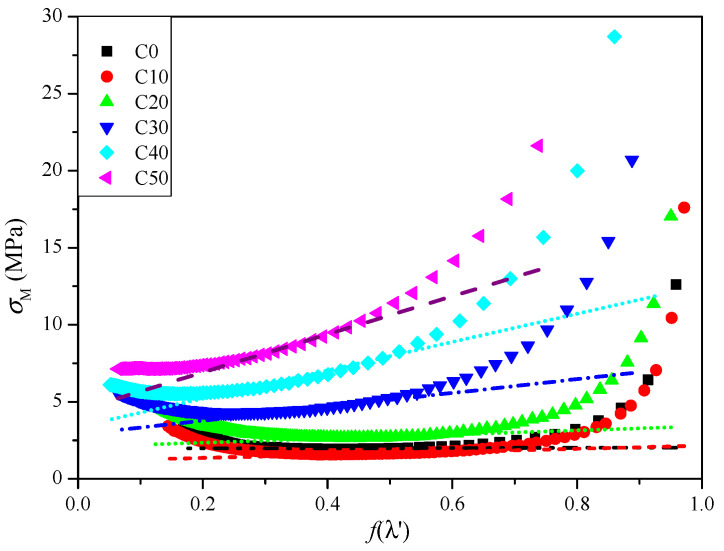
*σ_M_*–*f*(λ′) curves of CSDPF/NR vulcanizates.

Figure 4 is a *σ_M_*–*f*(*λ*′) diagram of CSDPF/NR vulcanizates with different contents of filler. It can be seen that under low strain (high *f*(*λ*′)), the *σ_M_* of all samples falls sharply, which is attributed to the Payne effect [11,14]. Under low strain, the network structure of the filler aggregates is destroyed, resulting in a significant reduction in the modulus of the vulcanizate; that is, there is a sharp decrease in *σ_M_*. However, under high strain (low *f*(*λ*′)), *σ_M_* of the samples shows a trend of increasing sharply; this is due to the tensile-induced crystallization of NR under high strain [15,16]. However, the trend is slow with the increase in CSDPF. This is attributed to the decrease in crystallization properties of NR with presence of filler [17].

In the middle area of Figure 4, the tangent line is cut along the flat area of the curve; the tangent intercept and the slope obtained correspond to the *G_c_* and *G_e_* of the vulcanizate, respectively. The results are listed in Table 3. It can be seen that the *G_c_* of the C10 sample is reduced and the *G_e_* increases upon adding a small amount of CSDPF. Compared with the unfilled vulcanizate (C0), it seems that the chemical crosslink part decreases in the vulcanization network of the C10 sample and the physical crosslink part increases. As mentioned above, the silica phase on the surface of CSDPF will adsorb the accelerators and reduce the degree of chemical crosslink of the rubber, so the *G_c_* of the vulcanizate is reduced. The rubber chain is entangled on the surface of CSDPF, which is the physical adsorption in bound rubber, and forms physical crosslinks [18]. Consequently, the *G_e_* of vulcanizate increases. As the filler increases, both the *G_c_* and *G_e_* of the CSDPF/NR vulcanizates are increased. For *G_c_*, on the one hand, more CSDPF causes greater interaction between the silica phases of CSDPF, and reduces the adsorption of accelerators to a certain extent. On the other hand, the chemical crosslink formed by the active point of filler and rubber segments [19] increases gradually, eventually leading to an increase in *G_c_*. For the *G_e_*, as the CSDPF increases, not only do the rubber chain segments adsorbed on the CSDPF surface increase, but the rubber segments will also be trapped in the CSDPF aggregates (inset image in Figure 2a). Therefore, *G_e_* increases.

The total crosslink network *G_c_* + *G_e_* obtained by the tube model and the data 1/$T_{2}^{XL}$ obtained by NMR are further compared with the XLD obtained by the swelling method; the data are listed in Table 3. Because the spin–spin relaxation time of NMR is inversely proportional to the crosslink density [20,21], the crosslink density of NMR method is replaced by 1/$T_{2}^{XL}$, accordingly. It can be seen that with the increase in filler, the trend of the crosslink network obtained by the tube model is consistent with that of the crosslink density obtained by the swelling method, which are all down first and then increasing, and the NMR method is not consistent with the above two: the 1/$T_{2}^{XL}$ is increasing with the increase in CSDPF. This is because the 1/$T_{2}^{XL}$ of NMR is only the characterization of crosslink degree in the vulcanization network; the tube model is more concerned with the crosslink situation of the rubber molecular chain on the surface of the filler. For the swelling method, the vulcanization network, the filler–rubber interaction and the filler–filler interaction are included [11]. The emphasis of the three methods is different; studying the structure of the rubber network from different angles is conducive to understanding the structure of the rubber network deeply.

## 4. Conclusions

CSDPF/NR vulcanizate was prepared by mechanical blending and the effects of CSDPF on the filler–rubber interaction and structure of rubber networks were studied. With an increase in CSDPF, the filler–rubber interaction in the compound increases. CSDPF can promote the formation of poly-sulfidic crosslinks in rubber vulcanization networks. Furthermore, the molecular chain movement ability of vulcanizates decreases and the crosslink density of vulcanizates increases, while the chemical crosslink and physical crosslink in vulcanization networks increase gradually.

## Figures and Tables

**Table 1:** Formulation of the CSDPF/NR compounds.

Sample	Constituent (Phr, Per Hundred Rubber)	
	CSDPF	NR
C0	0	100
C10	10	100
C20	20	100
C30	30	100
C40	40	100
C50	50	100

Other agents: ZnO 4, Stearic acid 2.5, Antioxidant RD 2.0, Coumarone indene resin 3, sulfur 2.0, accelerator NS 1.5.

**Table 2:** ^13^C-NMR data of CSDPF/NR vulcanizates.

Sample			C0	C10	C20	C30	C40	C50
Signal-to-noise ratio			1105	1009	1053	1130	1081	981
Relative intensity of signals (×100)	Mono-sulfidic crosslink	44.7 ppm	3.5	2.1	3.6	2.1	2.4	3.6
	Poly-sulfidic crosslink	44.1 ppm	3.5	2.3	3.8	2.3	2.6	3.5
		50.4 ppm	3.1	2.2	3.2	1.8	2.1	3.2
		58.0 ppm	2.8	1.7	2.9	1.6	1.8	3.5
Mono-sulfidic crosslink (%)			27.2	25.9	26.9	27.3	27.0	26.0
Poly-sulfidic crosslink (%)			72.8	74.1	73.1	72.7	73.0	74.0

**Table 3:** Parameters of network in CSDPF/NR vulcanizates.

Sample	*G_c_* (MPa)	*G_e_* (MPa)	*G_c_* + *G_e_* (MPa)	$1/T_{2}^{XL}$ (10^−2^ ms^−1^)	XLD (10^4^ mol·cm^−3^)
C0	2.0	0.7	2.7	4.73	4.32
C10	1.2	1.0	2.2	5.36	3.78
C20	2.1	1.3	3.4	5.87	4.64
C30	2.9	4.5	7.4	6.38	5.40
C40	3.4	9.2	12.6	6.57	6.08
C50	4.4	12.4	16.8	7.21	6.33

## Data Availability

Correspondence and requests for materials should be addressed to J.W.

## Abbreviations

CSDPF	Carbon–silica dual-phase filler
NR	Natural rubber
ZnO	Zinc oxide
RD	Poly(1,2-dihydro−2,2,4-trimethyl-quinoline)
NS	N-tert-Butyl-2-benzothiazolesulfenamide
XLD	Crosslink density
NMR	Nuclear magnetic resonance
MAS	Magic-angle spin
*T*_2_	Spin–spin relaxation time
CPMG	Carr–Purcell–Meiboom–Gill
BdR	Bound rubber
*G_c_*	Contribution of chemical crosslink to the elastic modulus
*G_e_*	Contribution of physical crosslink to the elastic modulus
